# On the Treatment of Dropsical Affections, by Injections of Iodine, &c

**Published:** 1850-10

**Authors:** 


### ECLECTIC DEPARTMENT,

### AND SPIRIT OF THE MEDICAL PERIODICAL PRESS.

---

*On the treatment of Dropsical Affections, by Injections of Iodine, &c.* By Daniel Brainard, M. D., Prof. of Surgery, in Rush Medical College.

In the Nos. of this Journal for Jan., 1848, and Feb., 1849, I related a case of Spina Bifida, treated by injections of Iodine and Iodide Potassium in solution.

The number of injections used in that case was fifteen, and the time required for the cure ten months, viz.: from December, 1847, to October, 1848. The strength of the solution used varied from ½ gr. Iodine, and gr. j. Iode Pot. to the f. oz. Dist. Water to gr. jv. Iodine, and gr. vj. Iod. Pot. to the f. oz. of water.

The cure was perfect and permanent, the patient remaining in good health to the present time.

This, the first of my published cases, was not, by any means, the first case of dropsical effusion in which I had used injections. As early as Dec., 1845, I threw into the peritoneum gr. xv. Iod. Potass, dissolved in f. oz. j. dist. water, after having, as perfectly as possible, evacuated the fluid, by tapping. An acute, smarting pain was produced, which subsided in a few minutes, and no sign of inflammation followed. The patient returned home, and I lost sight of him.

Since that time, Dr. F. Hagemann, of this city, injected the peritoneum of a dropsical patient twice, at my request. I have not notes of the case; it was one of incurable organic disease, but the injections produced no inflammation.

During the past winter I injected the abdomen of a patient laboring under general dropsy, as well as ascites from disease of the heart, with gr. jv. Iodine and gr. viij. Iod. Pot., in solution without drawing off the fluid. It was followed by no signs of inflammation, but the fluid in the peritoneum was absorbed, and a great amelioration of symptoms followed.

These were all supposed to be hopeless cases of disease in which the injection was used, not with the expectation of cure, but to ascertain the danger which there might be of producing inflammation. The result, in that respect, was perfectly satisfactory. I can now also add two other cases treated in a similar manner; one by Dr. M. Clure, of Dundee, Ill., and another by Prof. Mussey. These gentlemen have stated to me that they have recently used the injection without inflammation resulting. In the Am. Journal of Medicine, for Oct., 1847, p. 491, is the report of a case of ascites, cured by Mr. Leriche, of Lyons, in this manner. The injection was strong, and made, in this instance, after all the fluid had been drawn off.

This practice seems to have been tried with success a long time ago. Pereira speaks of it, and refers to the philosophical transaction of 1744.

Samuel Cooper, in his Surgical Dictionary, says he has seen two fatal cases from the practice. As they were both, as far as can be judged, from the injection of alcohol, after the fluid was evacuated by tapping, it is only surprising that one of the patients should have survived for a long time. Such cases do not authorize us to reject the practice when properly conducted, or justify the conclusion that it is necessarily dangerous.

It may, then, be considered an established fact that injections of this kind may be made, with suitable care, without danger of producing inflammation. It remained to be shown that dropsical effusions could be cured without such inflammation or adhesion of the sac in which it was situated.

A man applied to me during the past winter, with a large hydrocele, of eighteen months' standing. Injected f. dr. j. of a solution of Iod. Potas. and Iodine, gr. j. of the latter to gr. xij. of the former, to the oz. of water, without drawing off any fluid, the solution being thrown in through an exploring canula. This was subsequently repeated twice, and at the end of six weeks scarcely any trace of effusion remained, the man having continued his labor, and felt but slight tenderness in the organ.

Three cases of Spina Bifida have recently fallen under my care, all associated with hydrocephalus, in a greater or less degree, and rendered, therefore, unfavorable for perfect cure. I did not, on this account, neglect the opportunities they offered to test the effect of the Iodine treatment.

Chicago, April 12, 1849.

Case I.—I was called to see an infant, delivered by a midwife about an hour previously, affected with a Spina Bifida, situated upon the lower part of the lumbar division of the spine, which had been ruptured during labor. It seemed to have been about the size of the closed fist. At the point where it was ruptured, the sac was formed by little else than the membranes, being almost transparent over a space of one inch in length and two lines in breadth. The child was cold, and breathed with difficulty. I laid open the sac to the extent of the transparent part of its coverings, and saw the opening into the spinal canal, which was small—not large enough to admit the point of the little finger. Folding the collapsed sac together, I placed a compress upon it, and a band around the body, and directed the child to be warmed by artificial heat.

13th. Found the serum which had escaped and dried upon the compress, had closed the opening, and that the sac was distended with serum. Child warm, and has taken food.

14th and 15th. Remains in the same state.

16th. The sac has opened and discharged serum slowly. Child shows signs of spasmodic action. I re-opened the sac, and touching the surface around the opening into the spinal canal, with Nit. Arg., again folded the sides together and applied a compress, and bound it with as much force as was judged safe.

17th. Sac much inflamed; discharge of reddish serum. Child restless and feverish, but takes the breast. The discharge continued colored slightly, and gradually became offensive till the 25th, when I injected the sac with the following solution: Iodine, gr. jv.; Iod. Pot., grs. xij, to the oz. of distilled water, most of which escaped, but some was doubtless retained by the compress.

27th. Discharge less offensive and yellowish; sac much contracted, and of a deep red color. Child appears well, and takes the breast freely.

30th. Injection of oz. ss. of sol., as before; discharge purulent; less offensive.

May 2d. Injected it with sol. Sul. Copper, gr. j. to the oz. of water. Child well; discharge thin and purulent.

May 4th. Repeated the same. Child well; tumor subsiding.

6th. Repeated the injection.

May 12th. Opening closed. Child appears in health; head enlarged—bones separated.

During the whole of this time the child grew, and seemed in perfect health until

June 1st. Child died suddenly, of convulsions.

This case undoubtedly resulted fatally, from closing the fistulous opening in the back too soon, but it illustrates, in a striking manner, the favorable effect of injections into the spinal canal, and the little danger of inflammation to be apprehended from their use.

I was not at the time aware of the danger of keeping compresses over such an opening, and held, in common with the whole profession, the erroneous opinion that the danger in cases of inflammation of the meninges of the brain and spinal cord was greatest where an opening existed.

This is an error which has recently been pointed out by Dr. Thompson, of Columbus, Ohio. Far from closing such an opening, the sac should be punctured in cases of Spina Bifida, when violent inflammation of the membranes results from treatment and injections used, if the discharge is profuse and offensive.

Case II.—Spina Bifida, complicated with Hydrocephalus, treated with injections of Iodine.

Feb. 12, 1850. Was called to see a female child, three months old, born at seven months, excessively feeble at birth, and affected with Hydrocephalus and Spina Bifida. The tumor occupied the lumbar region; was 3½ inches in length, 2½ in breadth, and 1½ in elevation; elastic, fluctuating, semi-transparent.

I made a puncture with an exploring trochar 2 lines from the base of the tumor, and without drawing off the fluid injected 2-3 of a dr. of the following solution: Iodine gr. j.; Iod. Pot., gr. iij.; Aq. Dist., f. oz. j.; making 1-12 gr. Iodine, and ¼ Iod. Pot. thrown in.

Feb. 13th. Tumor tense and hot; pressure on it produces twitching of the limbs. Directed the child to be laid upon its face, and evaporating lotions applied to the part.

14th. Child appears well; tumor tense.

17th. Tumor flaccid, and reduced one-fourth in size. Injected 1-6 gr. Tannin in 2-3 f. dr. j. Dist. Water.

18th. Child restless and feverish; tumor hot, tense. Applied evaporating lotions, and gave a purge of Castor Oil.

19th. Child appears well. Applied solution of Gun Cotton to tumor.

March 2d. Tumor which had been flaccid and greatly reduced in size, commences to increase. Injected Sol. Tannin, as before, and with the same result.

7th. Injected gr. ss. Iodine, and gr. j. Iod. Pot. in f. dr. j. Distilled Water. But slight symptoms of inflammation resulted. Tumor diminishing rapidly in size, and becoming firmer, and of a flesh color.

10th. Repeated injection. Soon after the injection, twitching of the members occurred; the solution was therefore drawn off, and the sac partially filled with Dist. Water. The twitching immediately subsided. The child took the breast, and appeared in its usual health, and continued so for eighteen hours. At the end of that time, in the night, the mother noticed the child to be unwell, and, lighting a candle, found it in convulsions, of which it died. Such was the account given by the parents, who were sleeping in the bed with the child. Some circumstances prevented me from giving implicit credit to the account.

Prof. Davis assisted at the examination of tumor, twelve hours after death. The opening into the spinal canal was entirely closed. The sac contained f. dr. j. clear serum; its walls were thick and firm, without any effusion of lymph or unnatural vascularity. The head was tense and had increased rapidly in size for a few days before death. Dr. Davis coincided with me, in the opinion that there was no sign of irritation about the tumor, to account for the death. On the contrary, the effect of five injections in four weeks, in diminishing the size of the tumor, thickening its walls and closing up the opening into the spinal canal, without symptoms of inflammation, furnish the best evidence which could be desired of the efficiency and safety of the treatment. The Tannin was twice substituted for the Iodine solution, under the supposition that its astringency would be more effective in preventing effusion; it was found, however, to produce more inflammation, with less tendency to diminution afterward, and was therefore discontinued.

It may be stated that, from the time of the first injection, the child, which had before been puny, began to thrive, and grew rapidly up to the time of its death.

III.—Case of Spina Bifida and Hydrocephalus, treated with Iodine injections.

Sept. 20, 1849. Saw a female child of Mr. R., aged 4 days, affected with cleft spine and Hydrocephalus.

The cleft of the spine was situated at the lower part of the lumbar region, was about 3 inches in length and $1\frac{1}{2}$ in breadth; the abdomen was prominent, particularly on each side, the divisions of the spinal column, movable, showing that the want of union was not confined to the processes, but extended to the bodies of the vertebræ.

There was a fluctuating tumor situated over the fissure, about $\frac{3}{4}$ of an inch above the surface of the surrounding skin. Its covering was then of a blueish color, semi-transparent, and seemed composed of the thin membranes of the spinal cord, and the cuticle alone. Indeed, this latter was wanting over a considerable surface, where an ulceration of the size of a shilling existed.

The head was of large size, measuring 19 inches around the frontal and parietal protuberances. The upper part of the parietal and occipital bones was wanting, and the head bulged out into a very irregular, tense, and perfectly elastic sac. The other bones were imperfectly ossified and separated. The head was hot, covered with large veins, and increased rapidly in size. The right foot was affected with a Talipes varus, and the movement of the lower extremities was feeble.

The urine dribbled away, and excoriated the skin extensively.

Applications of Nit. Arg., in solution, were directed for the ulcerated spot until it was nearly healed. Cold water was applied to the head to keep down the heat.

Oct. 9, at 12½ o'clock, the treatmennt was commenced as follows:—

A very minute puncture was made with the point of the lancet, through the skin, half an inch below the tumor of the back, and a small sized exploring trochar passed from it obliquely upward into the sac. Without allowing the escape of more than a few drops of fluid, f. dr. ss. of a solution of Iodine, of the following strength was injected. Iodine, gr. j. Iod. Pot., gr. ij.; Aq. Dist. f. oz. j. Making 1-16 gr. of Iodine and ⅛ gr. of Hyd. Potas. in the injection. The puncture and surface of the tumor were then covered with a solution of Gun Cotton. No pain was felt except at the moment of puncturing the skin, and from the application of the liquid plaister. I was assisted by Professors Evans and Herrick, and Dr. Meek, editor of the N. W. Journal of Medicine.

10th. 12 o'clock, M. Found the child had been restless from the time of the operation until 6 o'clock, P. M., when it became quiet; seems well. Veins of head less turgid than since its birth.

11th. Child appears well; head less tense. Applied adhesive liquid to tumor and continued cold water to head.

12th. Head tense; increased in size ½ inch. Tumor of back flaccid, flattened and slightly inflamed.

13th. Tumor of back diminishing; head increasing rapidly. Ulceration of back quite cicatrized.

In consequence of the rapid increase in the size of the head, I determined to inject it without waiting, as I had intended to do, for the cure of the back to be completed. This was accordingly done with the assistance of Drs. Evans and Rutter. The point chosen was that most projecting on the summit and left side of the head.

About ½ dr. of fluid was allowed to escape. The same quantity of the solution (of the strength before given) injected, and the puncture covered with adhesive solution. Child seemed to suffer no pain except from puncture and application of ether upon the point.

14th, 15th, 16th. Head hotter, and increased in size; appears well.

16th. 8 o'clock, P. M. The puncture of head commenced leaking, which was stopped by Dr. Herrick with collodion.

17th, at 4 o'clock, A. M., leaked again, and was stopped by a compress and bandage. Head flaccid; child irritable, sleepless, and starting.

19th. Head filling. To avoid danger of leaking from former puncture, 1½ oz. of fluid was drawn off by a very oblique puncture on the right side.

20th. Child restless; affected with apathæ. Head hot.

21st. Child appears well; head natural.

31st. Head, which was stationary, has commenced to increase; measures 20 inches in circumference. It was injected at 5 o'clock, P. M., with f. dr. jss. of the solution. The puncture was made obliquely upon the summit of the right side.

Nov. 1st. For 24 hours, no effect was perceptible. At the end of this time, it commenced to be restless and sleepless, twitching often, and declined the breast. Evaporating lotions were applied to the head; a cathartic of Castor Oil administered.

2d. The child was much relieved, and took the breast. Head stationary—the whole of the back part of it covered with a red blush which had gradually spread from the puncture.

3d. Child restless. Head hot and red at the back part.

4th. Heat less; redness almost gone; head diminished in circumference, 3-4 inch in 24 hours.

5th. Child restless; head flaccid, diminished in size. Applied a band of India rubber, 4 inches broad, about it, to compress it.

6th. Has slept well from the time of application of band. Head diminishing.

7th. Continues to diminish. India rubber exchanged for a band of cotton.

From 7th to 24th, pressure continued; little change in head; remains stationary, at about 19 inches in circumference.

24th. Threw in 3-4 of a dr. of solution; no effect.

Dec. 2d. Injected f. dr. jss. of solution. Head increasing; no effect.

15th. Head soft; diminished in size $\frac{1}{2}$ inch in 24 hours.

At the first injection, but little fluid was allowed to escape. It was transparent, and showed, under the microscope, some epithelium cells. At the last operation, about one oz. was withdrawn; it was of a straw color; gave a slight cloud on application of heat, and was gelatinous on evaporation.

18th. Injected f. dr. jss. of the following solution: Iodine, iij.; Iod. Pot., gr. vj.; Aq. Dist., oz. j.; no effect.

30th. Threw in f. dr. jss. of the following solution: Iodine, grs. jvss.; Iod. Pot. gr. xij.; Aq. Dist. oz. j. No effect.

1850, Jan. 5th. Head is increasing rapidly; measures 22 inches in circumference.

Injected gr. ij. Iodine; grs. v. Iod. Pot. in f. dr. ij. Dist. Water. The fluid drawn off this time amounted to oz. iij.; was clear: exhibited, on standing, a filamentous appearance, like an exceedingly slight coagulum, and yielded, with starch, no trace of Iodine.

9th. Child was restless, and head slightly hotter than usual, after the last injection.

12th. Child well; head flaccid. Drew off oz. xij. fluid, and injected the same quantity as before, of the solution.

15th. Child slightly restless after injection, but soon became quiet, and exhibited no effect except a free secretion of tears and mucus from the eyes.

18th. Injected gr. jv. Iodine, and Iod. Pot., gr. x. in f. dr. vj. water.

19th. No effect except the secretions from the eyes.

22d. Child in perfect health; appears unusually bright, and is very active, moving its hands actively, and its feet slightly. Tumor of back reduced to a level with the surrounding skin. Urine retained naturally, and the excoriations healed.

26th. Drew off oz. xvj. fluid, and injected gr. viij. Iodine, and gr. xxjv. Iod. Pot., in water, f. oz. j.

Scarcely any effect was produced by this injection. The head was stationary for about four days after the injection, and then gradually increased. Compression by adhesive straps, bandages and Gun Cotton were tried perseveringly, without effect. Head 24 inches in circumference.

February 4th. Drew off f. oz. xxjv., and injected gr. xij Iodine, and gr. xxxvj. Iod. Pot. in oz. j. water. No effect was produced at the moment; but in the evening, 12 hours after the injection, there was twitching of the limbs and vomiting. The matter vomited colored the starched dress of the child of a dark color. The dress, diapers, bed and handkerchief used on it were impregnated with the Iodine odor for about three days after the injection, but the most careful tests, by Prof. Blaney, with starch, after adding chlorine water, never detected any trace of Iodine in the fluid drawn from the head.

19th. For several days the head remained stationary, but has commenced to enlarge. Injected gr. x. Iodine, with gr. xxx. Iod. Pot., after evacuating oz. xxjv. fluid. Head in this and other cases, when much fluid was drawn, supported with adhesive straps and bandages.

Effect the same as in the last instance; cold lotions were applied to the head, and purge of Castor Oil given.

March 5th. Head increasing. Drew off xxjv. oz. fluid, and injected the same quantity as before. Applied a laced cap to the head.

The symptoms resulting from this injection were but slight.

9th. Drew off vj. oz. fluid, and repeated the same injection. This quantity reduced the head to the same size as before, by which it appears that 1½ oz. serum formed daily.

*Remark.* During the treatment for the last two months, the child has grown rapidly; the face, which was triangular and thin, has filled up, is fat and firm; the skin, from being pale, is become ruddy; the limbs are firm, and the skin of the head, from having been glistening, smooth, with but little hair and large veins ramifying over it, is covered with a thick growth of hair, and appears very natural; the bones of the opposite sides touch, and the head is 20 inches in circumference; the bones being thick and firm. The tumor of back is entirely obliterated, and the skin in its former position becoming firm and of natural color.

10th. No effect; head paler than natural.

11th, 12th. Child restless.

15th. Is in good health. Drew off f. oz. vi., and injected gr. vij. Iodine, with gr. xxj. Iod. Pot. in oz. j. of water.

20th. No effect from last injection; used gr. x. Iodine, with thrice the quantity Iod. Pot. in the oz. water. Fluid drawn, oz. vj. A few bubbles of air probably passed in with the injection.

21st. Has had twitching. Head pale.

27th. Drew off the same quantity, and injected as before.

28th. Little effect; dress colored by matters vomited.

April 3d. Repeated the injection with gr. xij. Iodine, and gr. xxxvj. Iod. Pot. to the f. oz. of water. It produced vomiting and purging of matters which colored the clothing. It is probable the materials used were not pure, as they were not procured from the usual shop, and had a different effect from any former injection. Since the first commencement of the treatment it had been noticed that immediately after tapping, or while the head was diminishing in size, the child was wakeful, nervous, and affected with muscular twitching, on the slightest cause, while, on the contrary, it was quiet and had a tendency to sleep, when the head was full and increasing in size. Recently this tendency to somnolence has greatly increased—so much so that it could be at times scarcely roused. It was evident the disease was approaching its natural termination.

11th. Threw in gr. ix. Iodine, with gr. xxvij. Iod. Pot. in f. oz. j. Dist. Water, after withdrawing oz. vj. liquid. Slight twitching of the limbs the next day.

17th. Repeated the same quantity, after withdrawing f. oz. viij. liquid. After about 12 hours had elapsed, the child was affected with severe and dangerous symptoms, twitching, vomiting of matters which colored the clothing dark. These symptoms continued 48 hours, and were the worst the child had experienced since the commencement of the treatment.

The analogy supposed to exist between Spina Bifida and Hydrocephalus had at first given rise to the expectation that the treatment of the latter disease, by injections of Iodine, would be found efficient in its cure. This hope, after a fair trial, had in this case been abandoned, but the treatment was still continued, in the hope that its obvious effect in retarding the increase of the head might enable the bones to close at a size which would allow of health and intelligence in the child.

The gradually increasing severity of the symptoms, following each injection, and the tendency to somnolence and diminished activity and intelligence of the child, suggested the propriety of suspending their use.

Accordingly, on the 2d May, I drew off viij. oz. fluid, without any injection, the tension of the head and somnolence rendering such relief necessary. For two or three days, this afforded relief, but fluid accumulated with unusual rapidity, so much so that during my absence, in attendance on the National Medical Convention, at Cincinnati, the head attained a great size, the child became somnolent, and Prof. Herrick was called on to tap the head, which he did, May 12th, to the extent of vj. oz., with but partial relief.

It was in this condition that the head was found on my return, May 14. It was greatly increased in size, hotter than usual, covered with large, branching veins, and the child somnolent.

Notwithstanding the approaching fatal result of the case was apparent, yet the rapid aggravation of the symptoms under the treatment by tapping alone as compared with that by tapping and injection, induced us to resort again to this latter, as a palliation; accordingly, on the 15th, I threw in gr. vj. Iodine, with thrice that quantity Iod. Pot. in oz. j. water. The amount of fluid previously drawn off was oz. xij.

No perceptible effect was produced for 12½ hours, when the child commenced vomiting and purging, fell into a convulsion and died immediately.

### EXAMINATION AFTER DEATH.

The back, in the situation of the former tumor, was found smooth, firm, with but slight traces of disease. A cure of the Spina Bifida had evidently been effected.

On laying open the cranium, two-and-a-half pints of clear fluid was discharged. This was contained, principally, in the enlarged ventricles, there being but a thin layer on the surface. The arachnoid membrane was in a perfectly natural state, thin, polished, delicate; without any thickening or opacity. At the point where all the punctures but two had been made, the two layers of the arachnoid were closely adherent to each other. So that all the injections had been made into the ventricles, and not, as I had thought probable, into the sac of the arachnoid. The deception, in this respect, arose from the extreme tenuity of the ventricular walls, which over the upper part of the cerebrum were scarcely a line in thickness, and at points composed of the investing membranes of the organ, the cerebral substance having entirely given way, so that the canula could be easily felt, when in the ventricle, by the finger on the outside of the head.

Descending to the lower surface of the cerebrum, the walls of the cavity became thicker. The optic thalami, and corpora striata, although separated from each other, were entire, and, with the exception of flattening, in their natural state. The corpora quadragemina, medulla oblongata, and cerebellum, were well developed and healthy.

The internal surface of the immense cavity, was composed of white nervous tissue, firm and smooth, except at points where there were slight elevations, without any sign of lymph effused or unusual vascularity. Indeed, the whole substance of brain and its membranes, seemed less vascular than natural. The corpus callosum, extremely thin but not divided, formed the roof of the cavity. The *iter ad infundibulum* was dilated, but the *aquaduct of Sylvius* seemed not pervious without pressure. On the floor of the cavity lay the choroid plexus hypertrophied, and resembling a congeries of mucous follicles, rather than the natural condition of the membrane.

Between the cerebrum and the cerebellum was found a cyst of the size of a small plum, containing a serous fluid. On opening this, there was found a small surface, resembling entirely that of the choroid plexus in the large cavity.

This circumstance, together with the perfectly healthy appearance of the arachnoid, induced Prof. Herrick, who assisted me in this examination, to believe that the common opinion, that *internal* chronic Hydrocephalus originates from the arachnoid membrane, in so far, at least, as this case is concerned, is erroneous. He thinks the diseased choroid plexus was the source of the fluid. On examining that tissue with the microscope, his opinion (in which Prof. Davis concurred) was confirmed, for he found in this tissue, a great number of cells, arranged in groups, or in form of racemes attached to a common foot-stalk.

I am under the deepest obligation to Prof. Herrick, for assistance during the treatment of this case. I was also assisted by Profs. Evans, Blaney, Davis, Dr. David Rutter, and several other medical friends.

The inferences which I think may be legitimately drawn from these cases, are:

1. Injections into cavities affected with Dropsies, may be made without great danger; the violence of the symptoms will depend upon the strength of the injection, the quantity of the fluid present, and the duration of the disease. Old cases, when the membrane is thickened, or when the quantity of fluid is great, or when the surface has gradually become accustomed to the presence of the fluid, are those in which there is least danger to be apprehended.

2. Spina Bifida is generally a curable disease by such a treatment. Still I would by no means assert that success can be always expected. It is sometimes associated with mal-formations or incurable disease. The internal surface of the sac is often inlaid, in a great part of its surface, by the trunks of the sacral nerves. A puncture of one of these, whether in tapping, injecting or acupuncture, may be followed by serious results.

This frequent presence of nervous trunks, is a fatal objection to excision of the coverings of the tumor, to fetch the edges together by stitches.

In addition, Erysipelas or convulsions may occur in such cases, from a simple puncture.

Still, in general, I have no doubt the treatment will be found, as compared with any other known method, safe and efficient

The following are the circumstances to which I would particularly call attention as necessary to its safety and success.

Using not more than 1-32d part of a gr. Iodine, and three times as much Iod. Pot. at the commencement, dissolved in Dist. Water, and *be sure it is of perfect purity, and the solution recently made.*

As long as it produces moderate inflammation, do not increase the quantity.

Make the puncture 1-4th inch from the base of the tumor, in sound skin; it is much safer.

Do not evacuate the tumor, or, if it escapes, replace the fluid drawn off by Dist. Water.

If spasms should occur, the fluid might be withdrawn and Dist. Water injected.

The child should be laid upon its face, and if heat and tension occur, cold be applied to the part and head.

As soon as the tension is past, collodion should be applied and re-applied as long as it continues to diminish in size. When it ceases or increases, a new injection should be made.

3. The cases of ascites treated by me thus far, in this method, were such as offered favorable opportunities for testing injections in reference to the inflammation they might produce; it was necessary first to remove an erroneous opinion concerning their danger. I regretted, afterwards, not having continued their use, with the object of effecting a cure.

It is probable that inflammation has little or no connection with the cure of Dropsies by injection. It is only necessary, in such cases, to change the elements of the fluid, in some considerable degree, and the tendency to effusion ceases, the action of absorption becomes more rapid. It might be supposed that the case here narrated, of Hydrocephalus, militates against this opinion. We have already shown that it is most probable that the fluid in that case was the result of secretion from a morbid glandular structure in the choroid plexus, and not, by any means, an exhalation from the surface of the arachnoid membrane. If it were otherwise, and Hydrocephalus were analagous to ascites, anasarcha and other serous effusions, the want of success in curing it would not militate against its utility in other cases. To any one who saw the child at the commencement of the treatment, and observed the rapidity with which the head increased, there could be no doubt that, without treatment, the term of its life could be but a few days or weeks, at farthest. That the treatment cured the back, removed, in a great degree, the paralysis, and modified and retarded the progress of the disease in the head, will afford encouragement for applying it in any case less certainly fatal than that disease.

The effect of these injections, in filling and saturating every part of the system with the medicine, should not be overlooked. In the hydrocephalic child, the most violent symptoms followed effusion of the Iodine into the stomach. There was no salivation, as is the case when the medicine is thrown into the veins. Several years since, having occasion to treat a vascular tumor, of a doubtful nature, I threw into it a small quantity (the notes are not at hand) of Iodine and Hyd. Potash, in solution. In a few seconds the patient experienced a bitter taste in the mouth, and a free salivation which lasted a week. It was composed simply of enlarged veins, and the medicine had entered the circulation.

Convinced of this fact, the operation was repeated with the same result. In the case of injections into the head, on the contrary, the effect seemed to be to invigorate nutrition, and fatten the child, which grew, notwithstanding the effects following each operation. The importance of being acquainted with the action of other substances than those used thus far, when introduced into the cavities and tissues of the body, the effects they may produce in other diseases, such as ovarian cysts, anasarca, &c., might, with propriety be considered here. They will, however, naturally occur to the reflecting mind, and I shall defer their further notice until I can communicate some facts calculated to throw light on the subject.

CHICAGO, May 22d, 1850.

